# Proposed phase 2/ step 2 in-vitro test on basis of EN 14561 for standardised testing of the wound antiseptics PVP-iodine, chlorhexidine digluconate, polihexanide and octenidine dihydrochloride

**DOI:** 10.1186/s12879-017-2220-4

**Published:** 2017-02-13

**Authors:** Kathrin Schedler, Ojan Assadian, Uta Brautferger, Gerald Müller, Torsten Koburger, Simon Classen, Axel Kramer

**Affiliations:** 1 Institute of Hygiene and Environmental Medicine, University Medicine, Greifswald, Germany; 2 Institute for Sin Integrity and Infection Prevention, School of Human and Health Sciences, University of Huddersfield, R1/29 Ramsden Building, Queensgate, Huddersfield, HD1 3DH UK; 3 Hygiene Nord GmbH, Greifswald, Germany; 4 Department for Vascular Surgery, Kerckhoff-Klinik GmbH, Bad Nauheim, Germany

**Keywords:** PVP-iodine, Chlorhexidine digluconate, Polyhexanide, Octenidine dihydrochloride, EN 14561, Bactericidal efficacy, Yeasticidal efficacy, Wound antisepsis

## Abstract

**Background:**

Currently, there is no agreed standard for exploring the antimicrobial activity of wound antiseptics in a phase 2/ step 2 test protocol. In the present study, a standardised in-vitro test is proposed, which allows to test potential antiseptics in a more realistically simulation of conditions found in wounds as in a suspension test. Furthermore, factors potentially influencing test results such as type of materials used as test carrier or various compositions of organic soil challenge were investigated in detail.

**Methods:**

This proposed phase 2/ step 2 test method was modified on basis of the EN 14561 by drying the microbial test suspension on a metal carrier for 1 h, overlaying the test wound antiseptic, washing-off, neutralization, and dispersion at serial dilutions at the end of the required exposure time yielded reproducible, consistent test results.

**Results:**

The difference between the rapid onset of the antiseptic effect of PVP-I and the delayed onset especially of polihexanide was apparent. Among surface-active antimicrobial compounds, octenidine was more effective than chlorhexidine digluconate and polihexanide, with some differences depending on the test organisms. However, octenidine and PVP-I were approximately equivalent in efficiency and microbial spectrum, while polihexanide required longer exposure times or higher concentrations for a comparable antimicrobial efficacy.

**Conclusion:**

Overall, this method allowed testing and comparing differ liquid and gel based antimicrobial compounds in a standardised setting.

## Background and objectives

The potential antimicrobial activity can be tested following the European Norm EN 1040 (bactericidal activity) and EN 1275 (fungicidal and yeasticidal activity) [1, 2], both quantitative suspension tests without organic challenge. If a tested compound fails this phase 1 basic test, further investigations on its suitability as a biocide are futile. If, however, this phase 1 basic test is passed successfully, further tests simulating clinically relevant organic soil challenges (phase 2/ step 1 tests such as EN 13727 [3], a quantitative suspension test for the evaluation of bactericidal activity in the medical area) are required in order to assess the usability with simulated bioburden, which is recommended for application on mucous membranes with 10% albumin, 10% sheep blood, 1% mucin, or their combination, and for conditions representative for wounds with 10% albumin or 10% sheep blood challenge [4], or, maybe more representative for conditions on chronic wounds, Eagle’s Minimal Essential Medium (MEM) with Earle’s salts and L-glutamine, supplemented with 10% fetal calf serum without addition of antibiotics [5].

In the next step, so-called phase 2/ step 2 tests, standardised in-vitro carrier tests with and without organic soil exist for testing instrument disinfectants, e.g. EN 14561 bactericidal activity and EN 14562 (fungicidal and yeasticidal activity) [6, 7]. Yet, there is no agreed standard for exploring the antimicrobial activity of potential wound antiseptics in a phase 2/ step 2 test protocol.

Previous attempts to apply the test conditions of the European standards EN 14561 and EN 14562 for testing the antimicrobial efficacy of wound antiseptics on test disc carriers have successfully been conducted with medical honey and silver wound dressings [8, 9]. However, despite the general feasibility of disc carriers for testing wound antiseptics following a modification of the phase 2/ step 2 tests EN 14561 and EN 14562, currently there is no recommendation regarding the required effectiveness of wound antiseptics within the declared exposure time in the carrier test. To solve this problem, Ebert et al. have used a significant difference from the control as criterion for sufficient antiseptic effectiveness [9].

Considering the recommended efficacy for instrument disinfectants of ≥ 5 log_10_ or ≥ 4 log_10_ reduction within the declared exposure time against bacteria and yeasts (referred as reduction factor “RF”) [10], a plausible recommendation for wound antiseptics would be an log_10_ RF ≥ 5 without, and ≥ 3 with organic soil challenge. Aside of criteria for test interpretation, a number of other factors potentially influencing test results, such as suitability of different surface materials used as test carriers, the reproducibility of results using different antimicrobial compounds frequently used as wound antiseptics such as PVP-iodine (PVP-I), chlorhexidine digluconate (CHG), octenidine dihydrochloride (OCT), or polihexanide (PHMB), or selection of a reference compound as standard reference for future testing have not been explored. In the present study, the possible influence of such factors is investigated in detail.

## Materials and methods

The following test organisms were selected for all experiments: *Staphylococcus aureus* (ATCC 6538), *Enterococcus faecium* (ATCC 6057), and *Pseudomonas aeruginosa* (ATCC 15442). Testing of the influence of different carrier disc materials was conducted with *P. aeruginosa* only. Furthermore, OCT and PHMB were additionally tested against *Staphylococcus epidermidis* (ATCC 12228), *Serratia marcescens* (ATCC 13880), and *Candida albicans* (ATCC 10231).

PVP-I and CHG were tested as aqueous solutions made from poly(vinylpyrrolidone)-Iodine Complex 100% (CAS 25655-41-8, Sigma-Aldrich®) in a final concentration of 10% PVP-I, and 20% Chlorhexidine digluconate (CAS 18472-51-0, Sigma-Aldrich®). OCT and PHMB were tested as commercially available gels, manufactured by Schülke and Mayr GmbH (Norderstedt, Germany) under GLP conditions consisting of the following ingredients: 2.85% Glycerol 85%, 94.65% deionized water and 2.5% hydroxyethyl cellulose. OCT was tested in dilutions between 0.005, and 0.1%, PHMB was tested in dilutions of 0.02, 0.04, and 0.1%.

To determine the potential influence of different material used as test carriers, circular metal test carriers (diameter 20 mm, 1.2 mm thick) were made of stainless steel (1.4301 according to EN 10088–1, surface finish quality 2B on top and bottom according EN 10088–2; Cziotec GmbH, Greifswald, Germany), circular plastic carriers (diameter 12.7 mm, 3.0 mm thick) were made of polycarbonate (BioSurface Technologies Corp., Montana, USA), and for glass carriers, microscope examination slides were cut to a size of 26 mm × 26 mm (following ISO 8037/I; Glasbearbeitungswerk GmbH & Co. KG, Braunschweig, Germany).

For the production of microbial test solutions, a frozen bead with adherent organisms of the respective microbial test strain was taken from an overnight culture, transferred onto blood agar and incubated for 24 h at 36 °C ± 1 °C. For the final tests, 4 colony forming units (cfu) were dispersed in 30 mL of Eagle’s Minimal Essential Medium (MEM) with Earl’s salts and L- glutamine (PAA Laboratories, Germany) supplemented with 10% foetal bovine serum (FBS; Gibco) (MEM + FBS). 30 min prior to the start of a test, human erythrocytes were added to the suspension, yielding an organic blood soil of 30%.

The experimental setup was based on requirements of EN 14561 [6]. Disk carriers were placed in Petri dishes coated with 0.05 mL of test suspension and dried for 60 min in a workbench under laminar airflow. Then, 0.25 mL of test compound (solution or gel) was applied to the respective test carrier surfaces, assuring, that they covered the dried test suspension completely. Sterile distilled water or the basic gel without antimicrobial compound were used as negative controls in parallel. After 5 min, 30 min, 3 h, 10 h, and 24 h, the test carriers were placed in 10 mL test tubes (Greiner Bio-One GmbH, Frickenhausen, Germany), previously filled with 5 mL of sterile glass beads and 10 mL of neutralizing agent. Test tubes were then vortexed for 10 min. Serial dilutions of the resulting mixture were prepared in tryptone + 0.89% NaCl solution. Thereafter, volumes of 0.1 mL in at least two different dilutions were applied on Tryptone soy agar (TSA) in parallel. After 48 h of incubation at 36 °C ± 1 °C, the resulting colony forming units (cfu) were counted and the reduction factor (RF) was calculated according to the formula: log_10_ RF = lg (cfu1) - lg (cfu2), giving the log_10_ RF for the tested exposure time. Cfu1 denotes the number of cfu/mL not exposed to the sample (negative control), and cfu2 denotes the number of cfu/mL after exposure to the sample.

All neutralizing agents were tested and validated following EN 1040 and EN 1275. A mixture of 4% tween 80, 3% saponin, 0.4% lecithin, and 1% sodium dodecyl sulphate (SDS) in 1 l of distilled water was used as neutralising agent in experiments testing *S. aureus*, *S. epidermidis*, *E. faecium*, and *C. albicans*. For *P. aeruginosa* and *S. marcescens*, a mixture of 30 g tween 80, 3 g saponin, 1 g histidine, and 1 g of cysteine in 1 l of distilled water was used. To neutralise CHX lipofundin® MCT 20%, a mixture of soy bean oil, triglycerides, phosphatidylcholine, glycerol, α-tocopherol, sodium oleate and essential fatty acids (B.Braun Melsungen AG) was used.

## Results

Test suspensions spread out more evenly on glass test carriers and dried faster than on the metal or plastic disc carriers. Plastic disc carriers showed two more disadvantages: the relatively rough surface and the electric charges of plastic carriers caused the test suspension to form droplets with prolonged drying time. The uneven spread of the suspension across the plastic surface resulted in areas with a particularly high concentration of test bacteria. However, the recovery of test strains (*P. aeruginosa*) was better in plastic and metal carriers, while fewer test strains were recoverable from glass carriers. The most favourable test strain recovery was achieved from metal disc carriers (Table 1), with losses ranging at only 2 log_10_. Assessing these results it should be noted that *P. aeruginosa* begins to die off naturally after 3 h after transfer to dried test carriers. However, because the applied test solutions covered the metal disc carries evenly and well, and recovery of test strains was high, metal carriers were used for all further experiments.

**Table 1:** Recovery of *P. aeruginosa* from metal, plastic, or glass test carriers

Exposure time on test carrier	Metal carrier (9.4 log_10_)^a^	Log_10_ difference	Plastic carrier (9.5 log_10_)^a^	Log_10_ difference	Glass carrier (9.5 log_10_)^a^	Log_10_ difference
5 min	7.8 ± 0.1	1.6	7.9 ± 0.1	1.6	6.7 ± 0.1	2.8
30 min	7.3 ± 0.2	2.1	7.4 ± 0.1	2.1	6.6 ± 0.1	2.9
3 h	7.1 ± 0.1	2.3	7.0 ± 0.4	2.5	6.1 ± 0.2	3.4

^a^Initial cfu/mL count in the test suspension

The standardised test conditions allowed good comparison of different antimicrobial compounds with or without presence of organic soil challenges (Table 2).

**Table 2:** Mean ± standard deviations log_10_ RF of different liquid antiseptics with and without organic soil

Test compound Type of soil	Exposure time				
	5 min	30 min	3 h	10 h	24 h
10% PVP-I					
Without soil - *S. aureus*	5.4 ± 0.2	5.5 ± 0.1	5.7 ± 0.1	6.0 ± 0.1	4.6 ± 0.5
*E. faecium*	4.5 ± 0.7	5.1 ± 0.1	5.1 ± 0.1	n.g.	n.g.
*P. aeruginosa*	5.8 ± 1.3	6.3 ± 0.1	6.1 ± 0.1	6.2 ± 0.1	n.g.
MEM + FBS – *S. aureus*	4.6 ± 0.5	5.1 ± 0.1	5.0 ± 0.1	5.4 ± 0.1	5.5 ± 0.1
*E. faecium*	4.1 ± 0.6	4.6 ± 0.1	4.2 ± 0.1	n.g.	n.g.
*P. aeruginosa*	4.1 ± 0.4	6.0 ± 0.1	6.1 ± 0.1	n.g.	n.g.
CSL+ 30% blood – *S. aureus*	1.8 ± 0.7	4.2 ± 0.1	5.3 ± 0.1	6.2 ± 0.1	6.5 ± 0.1
*E. faecium*	1.3 ± 0.2	5.1 ± 0.1	5.1 ± 0.1	n.g.	n.g.
*P. aeruginosa*	2.3 ± 0.4	6.0 ± 0.1	6.1 ± 0.1	n.g.	n.g.
0.05% CHG					
Without soil - *S. aureus*	1.6 ± 0.2	3.7 ± 0.3	5.1 ± 0.8	5.8 ± 0.2	6.3 ± 0.1
*E. faecium*	0.2 ± 0.1	0.6 ± 0.2	1.2 ± 0.2	2.1 ± 0.2	2.6 ± 0.8
*P. aeruginosa*	0.1 ± 0.2	0.4 ± 0.4	2.1 ± 0.1	4.0 ± 0.7	3.0 ± 0.7
MEM + FBS – *S. aureus*	2.7 ± 0.4	3.0 ± 0.8	4.7 ± 0.2	5.0 ± 0.3	5.3 ± 0.1
*E. faecium*	0.5 ± 0.2	2.5 ± 0.4	3.6 ± 0.3	4.0 ± 0.1	4.8 ± 0.1
*P. aeruginosa*	0.8 ± 0.4	1.0 ± 0.2	5.4 ± 0.1	6.0 ± 0.1	6.6 ± 0.1
CSL+ *30% blood – S. aureus*	0.8 ± 0.1	1.3 ± 0.5	3.7 ± 0.4	5.9 ± 0.4	6.7 ± 0.2
*E*. faecium	0.3 ± 0.5	1.1 ± 0.3	1.8 ± 0.5	3.4 ± 0.7	4.6 ± 0.6
*P. aeruginosa*	0.1 ± 0.2	1.0 ± 0.3	4.7 ± 1.0	5.8 ± 0.9	7.1 ± 0.1
0.1% OCT					
Without soil - *S. aureus*	4.4 ± 0.1	4.5 ± 0.2	4.6 ± 0.2	5.2 ± 0.1	5.7 ± 0.2
*E. faecium*	1.5 ± 0.5	2.1 ± 0.4	4.7 ± 1.0	5.6 ± 0.1	5.1 ± 0.1
*P. aeruginosa*	1.0 ± 0.5	4.8 ± 0.4	4.8 ± 0.4	5.1 ± 0.2	6.2 ± 0.1
MEM + FBS – *S. aureus*	3.5 ± 0.5	3.4 ± 0.6	3.7 ± 0.6	4.2 ± 0.2	5.4 ± 0.2
*E. faecium*	2.5 ± 0.5	4.4 ± 0.4	4.4 ± 0.2	4.5 ± 0.3	4.3 ± 0.2
*P. aeruginosa*	1.1 ± 1.0	3.8 ± 0.3	3.8 ± 0.2	4.2 ± 0.1	5.7 ± 0.1
CSL+ 30% blood – *S. aureus*	1.2 ± 0.1	2.2 ± 0.1	5.5 ± 0.1	5.8 ± 0.1	5.9 ± 0.1
*E. faecium*	1.3 ± 0.1	2.6 ± 0.1	5.4 ± 0.1	5.4 ± 0.1	5.4 ± 0.1
*P. aeruginosa*	0.3 ± 0.1	0.8 ± 0.1	2.2 ± 0.1	6.0 ± 0.1	6.7 ± 0.1
0.05% OCT					
Without soil - *S. aureus*	3.2 ± 1.3	4.5 ± 0.2	4.6 ± 0.2	5.2 ± 0.1	5.7 ± 0.2
*E. faecium*	1.4 ± 0.3	2.3 ± 0.3	3.4 ± 1.8	5.6 ± 0.1	5.5 ± 0.1
*P. aeruginosa*	2.1 ± 1.1	4.8 ± 0.4	4.8 ± 0.4	5.5 ± 0.2	6.2 ± 0.1
MEM + FBS – *S. aureus*	3.5 ± 0.5	3.4 ± 0.6	3.7 ± 0.6	4.2 ± 0.2	5.4 ± 0.2
*E. faecium*	2.4 ± 1.2	4.4 ± 0.4	4.4 ± 0.2	4.5 ± 0.3	4.3 ± 0.2
*P. aeruginosa*	1.5 ± 0.1	3.8 ± 0.3	3.8 ± 0.2	4.2 ± 0.1	5.7 ± 0.1
CSL+ 30% blood – *S. aureus*	1.2 ± 0.1	1.4 ± 0.1	4.6 ± 0.2	5.8 ± 0.1	5.9 ± 0.1
*E. faecium*	1.1 ± 0.1	2.3 ± 0.1	5.4 ± 0.1	5.4 ± 0.1	5.4 ± 0.1
*P. aeruginosa*	0.2 ± 0.1	0.8 ± 0.1	1.8 ± 0.1	6.0 ± 0.1	6.7 ± 0.1
0.005% OCT					
Without soil – *S. aureus*	0.8 ± 0.9	4.5 ± 0.2	4.6 ± 0.2	5.2 ± 0.1	5.7 ± 0.2
*E. faecium*	0.5 ± 0.3	0.7 ± 0.3	1.4 ± 0.7	1.3 ± 0.3	2.9 ± 1.8
*P. aeruginosa*	0.4 ± 0.1	1.2 ± 0.4	3.1 ± 1.2	4.7 ± 1.1	5.8 ± 0.8
MEM + FBS – *S. aureus*	3.1 ± 0.7	3.4 ± 0.6	3.7 ± 0.6	4.2 ± 0.2	5.4 ± 0.2
*E. faecium*	0.9 ± 0.6	1.5 ± 0.5	2.5 ± 1.2	4.5 ± 0.3	4.3 ± 0.2
*P. aeruginosa*	0.9 ± 0.5	2.1 ± 1.1	3.8 ± 0.2	4.2 ± 0.1	5.7 ± 0.1
0.1% PHMB					
Without soil – *S. aureus*	2.4 ± 0.1	4.2 ± 0.3	5.4 ± 0.1	5.4 ± 0.1	6.1 ± 0.1
*E. faecium*	4.8 ± 0.2	5.1 ± 0.1	5.1 ± 0.1	5.2 ± 0.1	5.4 ± 0.1
*P. aeruginosa*	3.5 ± 0.1	5.5. ± 0.1	5.7 ± 0.1	5.8 ± 0.1	6.0 ± 0.1
MEM + FBS – *S. aureus*	1.3 ± 0.1	4.2 ± 0.3	5.6 ± 0.1	5.7 ± 0.1	6.1 ± 0.1
*E. faecium*	4.7 ± 0.3	5.2 ± 0.1	5.4 ± 0.1	5.4 ± 0.1	5.4 ± 0.1
*P. aeruginosa*	2.4 ± 0.1	5.2 ± 0.2	5.4 ± 0.1	6.1 ± 0.1	6.5 ± 0.1
CSL+ 30% blood – *S. aureus*	1.1 ± 0.1	1.7 ± 0.1	4.9 ± 0.4	5.8 ± 0.1	5.9 ± 0.1
*E. faecium*	2.1 ± 0.1	2.1 ± 0.1	4.8 ± 0.3	5.4 ± 0.1	5.4 ± 0.1
*P. aeruginosa*	0.2 ± 0.1	1.3 ± 0.1	5.8 ± 0.1	6.1 ± 0.1	6.8 ± 0.1
0.04% PHMB					
Without soil – *S. aureus*	1.3 ± 0.1	2.6 ± 0.1	5.1 ± 0.5	5.4 ± 0.1	6.1 ± 0.1
*E. faecium*	3.8 ± 0.1	5.1 ± 0.1	5.1 ± 0.1	5.2 ± 0.1	5.4 ± 0.1
*P. aeruginosa*	2.1 ± 0.1	4.5 ± 0.2	5.7 ± 0.1	5.8 ± 0.1	6.0 ± 0.1
MEM + FBS – *S. aureus*	0.8 ± 0.1	1.2 ± 0.1	5.6 ± 0.1	5.6 ± 0.1	6.1 ± 0.1
*E. faecium*	3.1 ± 0.1	5.1 ± 0.2	5.4 ± 0.1	5.4 ± 0.1	5.4 ± 0.1
*P. aeruginosa*	1.0 ± 0.1	3.4 ± 0.1	5.4 ± 0.1	6.1 ± 0.1	6.5 ± 0.1
CSL+ 30% blood – *S. aureus*	0.9 ± 0.1	1.2 ± 0.1	3.2 ± 0.1	5.8 ± 0.1	5.9 ± 0.1
*E. faecium*	1.5 ± 0.1	1.5 ± 0.1	2.7 ± 0.1	4.1 ± 0.1	5.4 ± 0.1
*P. aeruginosa*	0.1 ± 0.1	0.6 ± 0.1	4.1 ± 0.1	6.1 ± 0.1	6.8 ± 0.1
0.02% PHMB					
Without soil – *S. aureus*	−0.1 ± 0.1	0.5 ± 0.1	2.8 ± 0.3	5.2 ± 0.1	5.7 ± 0.2
*E. faecium*	0.3 ± 0.1	0.4 ± 0.2	0.4 ± 0.1	0.2 ± 0.1	0.1 ± 0.1
*P. aeruginosa*	0.4 ± 0.6	0.2 ± 0.3	1.2 ± 0.8	1.5 ± 0.4	1.4 ± 0.6
MEM + FBS – *S. aureus*	0.6 ± 0.4	1.7 ± 1.3	3.7 ± 0.6	4.2 ± 0.2	5.4 ± 0.2
*E. faecium*	0.5 ± 0.2	1.1 ± 0.3	1.1 ± 0.4	1.0 ± 0.9	1.6 ± 0.5
*P. aeruginosa*	0.5 ± 0.8	0.9 ± 0.1	3.0 ± 1.1	3.2 ± 1.8	3.8 ± 2.1
Controls (test strain: *S. aureus*)					
Gel	−0.1 ± 0.2	0.1 ± 0.4	0.3 ± 0.2	0.9 ± 0.3	1.4 ± 0.3
Gel + MEM + FBS	0.1 ± 0.2	0.3 ± 0.3	0.3 ± 0.2	0.8 ± 0.1	1.5 ± 0.6
Distilled water^a^	−0.1 ± 0.2	−0.1 ± 0.2	0.3 ± 0.4	1.0 ± 0.4	1.7 ± 0.6

^a^Control for aqueous solutions without soil

Ten percent PVP-I achieved the proposed required antiseptic efficacy of RF ≥ 5 without and ≥ 3 in the presence of simulated wound fluid within 5 min against *S. aureus* and *P. aeruginosa*, and against *E. faecium* within 5 min without and 30 min with organic challenge. Same results against *E. faecium* were observed with 0.05% (after 3 h) and 0.1% (after 30 min) OCT. Against *E. faecium*, PHMB achieved the required efficacy only at concentrations of 0.1 and 0.04%, whereas the lowest use concentration of 0.02% was not effective within 24 h.

Other tested antiseptics were more effective in the presence of simulated wound fluid, while blood reduced the effectiveness of all compounds. Interestingly, a dose/ exposure time depending influence was lowest for OCT; even at 0.005%, OCT achieved RF ≥ 5 without and ≥ 3 in the presence of simulated wound fluid within 5 min against *S. aureus* and *S. epidermidis*. However, 0.1% and 0.05% OCT were not as effective against *P. aeruginosa* as other tested compounds. In presence of organic soil, CHG was only effective against *P. aeruginosa* after 3 h, and performed better against *S. aureus* and *E. faecium*. The same was observed for PHMB, as in presence of soil, higher concentrations or longer exposure time were needed to reach a sufficient reduction. The concentration/ exposure time dependency of PHMB was remarkably seen even against *S. epidermidis*, where 0.02% PHMB require 10 h to be effective, but with higher concentrations the exposure time decreased sharply (Table 2).

*S. marcescens* showed the highest tolerability against the tested antiseptics compared to all other tested bacteria. In the presence of organic soil, 0.1% OCT as well as 0.1% PHMB were not able to achieve reductions at 30 min exposure time. At a concentration of 0.01% OCT, the required exposure time was increased to 3 h, and at 0.005% OCT to 10 h (Table 3). *C. albicans* remarkable tolerability against tested antiseptics compared to the tested bacteria. In presence of organic soil, OCT was effective at concentrations ranging from 0.1 to 0.02%, however, only after an exposure time of 24 h. Compared to OCT, PHMB showed a better antifungal efficacy. 0.1% PHMB was effective within 30 min in presence of organic soil, and at concentration ranging from 0.04 to 0.02% within 3 up to 10 h.

**Table 3:** Required exposure time (h) to achieve a log_10_ RF ≥ 5 (without soil) or log_10_ RF ≥ 3 (with organic soil challenge)

Test compound	Soil	*S. aureus*	*S. epidermidis*	*E. faecium*	*P. aeruginosa*	*S. marcescens*	*C. albicans*
10% PVP-I	Without	0.0833	Not tested	0.5	0.0833	Not tested	Not tested
	MEM + FBS	0.0833		0.0833	0.0833		
	Blood	0.5		0.5	0.5		
0.05% CHG	Without	3		>24	>24		
	MEM + FBS	0.5		3	3		
	Blood	3		10	3		
0.1% OCT 0.05% OCT 0.02% OCT 0.01% OCT 0.005% OCT	Without	10	0.0833	10	10	10	24
		10	3	10	10	>24	24
		10	3	24	10	>24	>24
		10	10	24	10	>24	>24
		10	10	>24	10	>24	
	MEM + FBS	0.0833	0.0833	0.5	0.5	0.5	24
		0.0833	0.5	0.5	0.5	0.5	24
		0.0833	0.5	0.5	0.5	0.5	>24
		0.0833	0.5	0.5	0.5	3	>24
		0.0833	0.5	10	0.5	10	>24
	Blood	3	n.t.	3	10	n.t.	n.t.
		3	n.t.	3	10	n.t.	n.t.
		n.t.	n.t.	n.t.	n.t.	n.t.	n.t.
		n.t.	n.t.	n.t.	n.t.	n.t.	n.t.
		n.t.	n.t.	n.t.	n.t.	n.t.	n.t.
0.15% PHMB 0.04% PHMB 0.02% PHMB	Without	3	0.0833	0.5	0.5	3	10
		3	0.0833	0.5	3	10	24
		10	>24	>24	>24	>24	>24
	MEM + FBS	0.5	0.0833	0.0833	0.5	0.5	3
		3	0.0833	0.0833	0.5	3	10
		3	10	>24	10	10	>24
	Blood	3	n.t.	3	3	n.t.	n.t.
		3	n.t.	10	3	n.t.	n.t.
		n.t.	n.t.	n.t.	n.t.	n.t.	n.t.

*n.t.* not tested

## Discussion

Chronic, and more so, acute wounds always exhibit organic matter and blood. Therefore, when applying antiseptics to wounds, the potentially inhibiting influence of such organic soil must be considered. In this respect, testing wound antiseptics without soil seems dispensable. Hence, we propose to employ only test conditions in the presence of soil relevant for wounds for future testing. Based on this aspect, only the results with organic soil are further discussed. Yet, not only presence or absence of organic soil matters, but also the type of organic challenge. Our results clearly demonstrate the influence of the selected challenging soil in terms of required concentrations or exposure time of various antiseptics. For instance, if 10% PVP-I is tested against *S. aureus* in presence of MEM + FBS, an exposure time of 5 min is required to achieve a ≥ 3 log_10_ RF. However, if the same test is conducted in presence of CSL+ 30% whole blood, 30 min exposure time are warranted to achieve the same antimicrobial effect. The same observation pertains to CHX, OCT, and at lower concentrations to PHMB (Table 2).

While conducting antimicrobial efficacy tests for wound antiseptics in presence of organic soil is plausible, selection of an appropriate organic soil surrogate is not trivial. Although MEM + FBS largely corresponds to physiological wound fluid as proposed by Campbell et al. previously [5], it does not simulate probable presence of blood. In addition to MEM + FBS, we therefore propose adding 30% blood to simulate a worst-case soil condition for further testing.

In many parts of the world, PVP-I is one of the most commonly used wound antiseptics because of its broad microbial spectrum and rapid onset of action [11–13]. Our results were able to support the view on these aspects. Indeed, even in presence of MEM + FBS, PVP-I was effective within 5 min against *S. aureus*, *E. faecium*, and *P. aeruginosa*. Furthermore, when challenged with 30% blood, the required exposure time was extended to not longer than 30 min. The negative impact of blood on PVP-I’s antimicrobial efficacy is explained by haemoglobin’s effect as an inhibitor of the antiseptic effect of iodine [4, 14]. Free-floating in a liquid environment, this effect may be even stronger, such as shown by Werner et al. [15], who used a quantitative suspension test at a 20% blood challenge. If, however, as in conditions present with the proposed disc carrier test, a sufficiently high PVP-I concentration is available on a dried surface, sufficient amounts of free iodine may overcome the chemical equilibrium, resulting in minimum prolonged exposure times, increasing from 5 to 30 min for the same antimicrobial effect. This situation corresponds to reality much better than in the suspension test because microbial suspension and antiseptic substance are only incompletely mixed when applied to the test surface, as well as to a wound. However, in reality, a mixture of both conditions may be present, depending on the exudation grade of a wound. Since PVP-I is characterized by good antiseptic and broad-spectrum antimicrobial efficacy with low variance of results, it could be used as reference compound in future for testing the efficacy of various antiseptics.

Based on our results, CHG, although also frequently used as an antiseptic globally, would not be a suitable candidate as reference compound because of its insufficient efficacy depending on the test species and in presence of bioburden. Even with the experimental setup being exactly replicated, the results varied by more than 2.5 log_10_.

Another suitable candidate would be OCT. In presence of MEM + FBS, OCT was bactericidal at concentrations of 0.1% within 5 min, and 0.05% within 30 min, depending on the test organism. Its efficacy against *S. aureus* is comparable to PVP-I, however, against *E. faecium* and *P. aeruginosa*, OCT required longer exposure times ranging between 30 min and 10 h. One of the remarkable features of OCT is that even when diluted to 0.005%, OCT remains effective against all tested bacteria after 10 h of exposure time. This corresponds well with other laboratory-based in-vitro and clinical in-vivo studies [13, 16, 17]. However, while OCT is widely used in Germany, Austria and Switzerland [16], it is not well known yet in many regions of the world.

Finally, PHMB requires longer exposure times than OCT against most tested microorganisms. This is in accordance with results obtained in previous quantitative suspension tests [13]. Low PHMB concentrations of 0.02% and below do not fulfil the antimicrobial requirement against *E. faecium* and *C. albicans*. However, at concentrations at 0.04% and if longer exposure times can be maintained, PHMB exhibits a number of positive features, including low cytotoxicity and no systemic resorption [18, 19].

One limitation of the here presented phase 2/ step 2 disc carrier test for wound antiseptics is that this test is not able to assess tissue tolerability. However, such certainly clinically important aspects are not the primary aim of a phase 2/ step 2 simulated in-vitro tests. In order to assess an antimicrobial effect and possible cytotoxicity, another test, which allows calculation of the “biocompatibility index” was presented elsewhere by Müller and Kramer [17]. Indeed, one reason for the increasing use of OCT and PHMB for wound antisepsis in Central Europe is their favourable biocompatibility index of > 1 and the associated lower cytotoxicity in comparison to PVP-I and CHG [17].

A second limitation pertains to the possibility that test strains may have reacted differently to drying and that the recovery from disk carriers considered only viable strains. However, all tests were comparable in terms of physical parameters such as drying time, exposure time, and recovery technique. *P. aeruginosa* begins to die after 3 h after being transferred to a dry surface. The preparation time for the test discs and recovery of the viable bacteria was notably shorter than 3 h. Therefore, a drying effect did not influence the obtained results of the recovery rates. Furthermore, since recovery focused on viable bacteria only in all tests, a possible sampling error may have occurred, which, however, would have been distributed evenly through all samples.

In conclusion, this adopted phase 2/ step 2 test method modified on basis of the EN 14561 by drying the microbial test suspension on a metal carrier for 1 h, overlaying the test wound antiseptic, washing-off, neutralization, and dispersion at serial dilutions at the end of the required exposure time yielded reproducible, consistent test results. This method allows testing and comparing differ liquid and gel based antimicrobial compounds in a standardised setting. Because PVP-I shows a rapidly deploying, stable antimicrobial effect relatively independent of external circumstances, it is proposed to be an ideal candidate used as standard reference in testing wound antiseptics.

## Abbreviations

ATCC: American Type Culture Collection (Manassas, VA, USA)
CFU: Colony forming unit
CHG: Chlorhexidine digluconate
EN: European Norm
FBS: Foetal bovine serum
GLP: Good Laboratory Practice
MEM: (Eagle’s) Minimal Essential Medium
OCT: Octenidine dihydrochloride
PHMB: Polihexanide
PVP-I: Povidone-Iodine
RF: Reduction factor
SDS: Sodium dodecyl sulphate
TSA: Tryptone soya agar
